# The impact of COVID-19 pandemic on nosocomial multidrug-resistant bacterial bloodstream infections and antibiotic consumption in a tertiary care hospital

**DOI:** 10.3205/dgkh000418

**Published:** 2022-08-29

**Authors:** Gökhan Metan, Mervenur Demir Çuha, Gülsen Hazirolan, Gülçin Telli Dizman, Elif Seren Tanriverdi, Baris Otlu, Zahit Tas, Pinar Zarakolu, Zafer Arik, Arzu Topeli, Seda Banu Akinci, Serhat Ünal, Ömrüm Uzun

**Affiliations:** 1 Department of Infectious Diseases and Clinical Microbiology, Hacettepe University Faculty of Medicine, Ankara, Turkey; 2 Infection Control Committee Hacettepe University Hospitals, Ankara, Turkey; 3 Department of Medical Microbiology, Hacettepe University Faculty of Medicine, Ankara, Turkey; 4 Department of Medical Microbiology, İnönü University Faculty of Medicine, Malatya, Turkey; 5 Division of Medical Oncology, Department of Internal Medicine, Hacettepe University Faculty of Medicine, Ankara, Turkey; 6 Section of Intensive Care Medicine, Department of Internal Medicine, Hacettepe University Faculty of Medicine, Ankara, Turkey; 7 Section of Intensive Care Medicine Department of Anesthesiology and Reanimation, Hacettepe University Faculty of Medicine, Ankara, Turkey

**Keywords:** COVID-19, SARS-CoV-2, antibacterial resistance, bacteremia, antibiotic consumption

## Abstract

We investigated the change in the epidemiology of nosocomial bloodstream infections (BSIs) caused by multidrug-resistant bacteria during Coronavirus Disease (COVID-19) and antibiotic consumption rates at a pandemic hospital and at the Oncology Hospital which operated as COVID-19-free on the same university campus. Significant increases in the infection density rate (IDRs) of BSIs caused by carbapenem-resistant *Acinetobacter baumannii* (CRAB) and ampicillin-resistant *Enterococcus faecium* (ARE) were detected at the pandemic hospital, whereas carbapenem-resistant *Klebsiella pneumoniae* BSIs were increased at the non-pandemic Oncology Hospital. Pulsed field gel electrophoresis showed a polyclonal outbreak of CRAB in COVID-19 intensive care units. Antibiotic consumption rates were increased for almost all antibiotics, and was most significant for meropenem at both of the hospitals. Increased IDRs of CRAB and ARE BSIs as well as an increased consumption rate of broad-spectrum antibiotics emphasize the importance of a multimodal infection prevention strategy combined with an active antibiotic stewardship program.

## Abstract (de)

Es wurden die Veränderungen in der Epidemiologie nosokomialer Blutstrominfektionen (BSI) durch multiresistente Bakterien und die Antibiotika-Verbrauchsraten während der COVID-19 Pandemie in einem mit an COVID-19 Erkrankten belegten Krankenhaus und der Onkologischen Klinik auf demselben Universitätsgelände ohne Belegung mit COVID-19 Patienten untersucht. Im Pandemiekrankenhaus wurde ein signifikanter Anstieg der Infektionsdichte (IDR) von BSI, verursacht durch Carbapenem-resistente *Acinetobacter baumannii* (CRAB) und Ampicillin-resistente *Enterococcus faecium* (ARE), festgestellt, während Carbapenem-resistente *Klebsiella pneumoniae* BSI in der nicht-pandemischen Onkologischen Klinik erhöht waren. Die Pulsfeld-Gel-Elektrophorese zeigte einen polyklonalen Ausbruch von CRAB auf den COVID-19-Intensivstationen. Der Antibiotikaverbrauch war bei fast allen Antibiotika erhöht, am stärksten bei Meropenem in beiden Krankenhäusern. Erhöhte IDRs von CRAB und ARE BSIs sowie ein erhöhter Verbrauch von Breitspektrum-Antibiotika unterstreichen die Bedeutung einer multimodalen Strategie zur Infektionsprävention in Kombination mit einem aktiven Antibiotic-Stewardship-Programm.

## Introduction

The Coronavirus disease (COVID-19) pandemic has significantly affected healthcare systems all over the world. Apart from the pandemic itself, collateral damage seems to be at least as important as the viral disease itself [1], [2], [3]. One of the most devastating examples of collateral damage of the pandemic is the increasing rate of multidrug-resistant (MDR) bacterial infections [4]. Although several hospitals reported a decrease in the rate of MDR bacterial infections during the early phase of the pandemic as a result of increased rates of compliance with hand hygiene and contact precautions [5], [6], a recent study from the USA reported a 3-to-5-fold increase in MDR bacterial bloodstream infections (BSIs) in hospitals serving COVID-19 patients with more than 300 beds [7].

The first patient with COVID-19 in Turkey was reported in March 2020, with a total of 9,364,508 cases of COVID-19 and 81,917 deaths as of December 29, 2021 [8]. Hacettepe University Adult Hospital has served as a pandemic hospital since the beginning of the pandemic, whereas Hacettepe University Oncology Hospital was designed to be a COVID-19-free hospital on the same campus. A total of 1365 patients with COVID-19 were hospitalized in COVID-19 wards and 316 in COVID-19 intensive care units (ICUs) between March 20, 2020 and November 20, 2020 at the pandemic hospital [9]. This study analyzed the trends in the rates of nosocomial BSIs caused by MDR bacteria and consumption rates of the intravenous antibiotics between 2018 and 2020 to investigate the impact of the COVID-19 pandemic. 

## Material and methods

Hacettepe University Hospitals are tertiary care centers in Ankara, the capital city of Turkey. Adult Hospital has 1,040 beds, including six ICUs with 143 beds. Oncology Hospital has 119 beds, including a 16-bed hematopoietic stem-cell transplantation ward and an 8-bed ICU. During the study period, blood specimens were drawn at bedside and cultured using the BACTEC 9240 blood culture system (Becton Dickinson, Cockeysville, MD, USA). Species of the bacteria that were isolated in blood culture were identified by Matrix-Assisted Laser Desorption/Ionization time-of-flight, Mass Spectrometry (MALDI-TOF MS). Antibiotic susceptibility tests were carried out and interpreted in accordance with the European Committee on Antimicrobial Susceptibility Testing (EUCAST) breakpoints [10]. 

The results of all blood cultures were reviewed to identify the number of MDR bacterial BSIs between January 01, 2018 and December 31, 2020. MDR bacteria were defined as extended spectrum beta-lactamase (ESBL) producing Escherichia coli (ESBL-EC), ESBL producing *Klebsiella pneumoniae* (ESBL-KP), carbapenem-resistant *K. pneumoniae* (CRKP), carbapenem-resistant *Pseudomonas aeruginosa* (CRPA), carbapenem-resistant *Acinetobacter baumannii* (CRAB), methicillin-resistant *Staphylococcus aureus* (MRSA), and vancomycin-resistant *Enterococcus faecium* (VRE). Although not MDR, we included BSIs caused by ampicillin-resistant *E. faecium* (ARE) in our analysis since they may trigger the increased consumption of glycopeptides. Only blood cultures that were drawn on hospital day 3 or later were included in the study. BSI with the same MDR bacteria after 14 days of the first episode was defined as a new episode. Infection density rates (IDR) of BSIs caused by MDR bacteria were calculated for 2018, 2019, and 2020 per 10,000 patient days; conditional maximum likelihood estimate (CMLE) of rate ratio (RR), 95% confidence intervals (CI) were calculated by using OpenEpi (Open-Source Epidemiologic Statistics for Public Health) version 3.01 (https://www.OpenEpi.com). The Mid-P exact test was used to compare CMLE in COVID ICUs with other ICUs as well as to compare CMLE in COVID-19 wards with other medical wards. P <0.05 was considered statistically significant.

A limited number of *A. baumannii* strains that were isolated from various clinical samples (blood 11, deep tracheal aspirate 2, and sputum 2 samples) taken from patients hospitalized at COVID-19 ICU on September 2020 underwent molecular epidemiological investigation by pulse-field gel electrophoresis (PFGE) as described previously [11]. Infection Control Committee reports were reviewed to identify the incidence rate of CRAB infections in COVID-19 ICUs. 

This report was prepared as a routine practice of infection control committee, and its publication was approved by Hacettepe University Non-Interventional Clinical Researches Ethics Board.

## Results

The IDR of BSIs caused by ESBL-EC, ESBL-KP, CREC, CRKP, MRSA, and VRE were similar each year. However, there was a significant increase in the IDRs of BSIs caused by CRAB and ARE in the pandemic hospital in 2020 compared to those IDRs in 2018 and 2019 (Table 1 (Tab. 1)). 

The IDRs of CRAB and ARE were higher in COVID-19 ICUs than non-COVID-19 ICUs in the pandemic hospital, whereas the IDRs of CRKP was higher in the latter (Table 2 (Tab. 2)). 

The highest IDR of CRKP per 10,000 patient days was detected in the Neurology ICU (44.05, 95% Confidence interval (CI) 23.8–74.8), followed by the Medical ICU (24.98, 95% CI 11.6–47.43), and the General Surgical ICU (19.6, 95% CI 7.2–43.6). The IDRs of BSIs caused by ESBL-KP, CRPA, MRSA, VRE, and ARE were similar in the pandemic hospital and Oncology Hospital; however, the IDR of CRAB BSI was higher in pandemic hospital and the IDR of CRKP and ESBL-EC BSIs were higher in the Oncology Hospital (Table 1 (Tab. 1)). There were no cases of carbapenem-susceptible ESBL-KP in COVID-19 ICUs.

**Figure 1:**
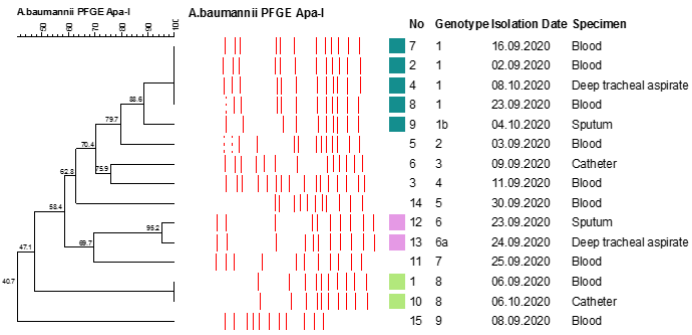
Clonal dispersion of *Acinetobacter baumannii* isolated from the patients at a COVID-19 intensive care unit in September 2020. PFGE analysis showed 9 genotypes in 15 strains isolated from 14 patients.

During the early phase of the pandemic, the IDRs of BSIs and pneumonia caused by CRAB were low in the first (5.47, 95% CI 2.01–12.2) and second quarter (2.95, 95% CI 0.75–8.02) of 2020, but there was a significant increase in the third (14.8, %95 CI 9.5-22) and fourth quarters (11.9, 95% CI 7.9–17.3). PFGE revealed nine different genotypes in 15 CARB strains isolated from various clinical samples obtained from 14 patients hospitalized at the COVID-19 ICU in September 2020 (Figure 1 (Fig. 1)). 

There was no difference in IDRs of BSIs between COVID-19 wards and other medical wards regarding ESBL-KP, CRAB, and ARE. The IDRs of BSIs caused by ESBL-EC and CRKP was higher in other medical wards than in COVID-19 wards. There were no cases with CRPA, VRE, and MRSA BSIs in COVID-19 wards (Table 2 (Tab. 2)).

Meropenem consumption almost doubled in 2020 at the pandemic hospital (Table 3 (Tab. 3)) with the highest consumption rate in COVID-19 ICUs followed by non-COVID-19 ICUs and the non-pandemic Oncology Hospital (Table 4 (Tab. 4)). There were also significant increases in the consumption rates of colistin, glycopeptides, and tigecycline in the pandemic hospital during the study period (Table 3 (Tab. 3)). There were slight increases in the consumption rates of ceftriaxone, amikacin, and piperacillin-tazobactam, while the consumption rates of ampicillin-sulbactam, ciprofloxacin, and ertapenem decreased at the pandemic hospital (Table 3 (Tab. 3)). 

The highest consumption rate for piperacillin-tazobactam was detected in non-COVID-19 ICUs (Table 4 (Tab. 4)).

## Discussion

We detected increased IDR of BSIs caused by CRAB in this study. This finding was more evident in the third quarter of 2020, concomitant with a sharp increase in the number of COVID-19 patients in Turkey. The number of ICUs beds was expanded rapidly in August 2020, and severely ill patients with COVID-19 were transferred to our pandemic hospital from other centers. The increased workload combined with a shortage of experienced staff, due to sick- or close-contact leave, may have resulted in a decrease in compliance with infection control protocols. Although molecular epidemiological investigation was limited to 15 strains that were isolated during the peak of CRAB infections in COVID-19 ICU, the presence of multiple genotypes (9 genotypes) in 14 patients indicates multiple sources for the spread of CRAB, instead of a single source for the outbreak (Figure 1 (Fig. 1)). Several CRAB outbreaks were reported from ICUs designed for COVID-19 patients [12], [13]. A recent study of 99 ICUs from Brazil showed a correlation between COVID-19 density and CRAB infections, but not CRKP and CRPA [14]. 

*Enterococcus spp*. was reported as the most common bacteria causing BSIs during a COVID-19 surge in the South Bronx, New York [15]. Contact precautions are strictly implemented in patients with VRE but not ARE. The increased rate of BSIs caused by ARE in our COVID-19 ICUs is worrisome because of the potential increase in VRE rates as a result of widespread glycopeptide treatment. A meta-analysis which included 19 studies and 20,304 patients found that VRE infection rate was 4.4 higher in patients who received vancomycin treatment [16].

A decrease in the rate of healthcare-associated infections was reported from the palliative care ward of the Oncology Hospital when the first three months of the pandemic were compared with the previous year [17]. However, there was an upward trend in IDR of BSIs caused by CRKP in the Oncology Hospital, whereas IDRs of BSIs caused by other MDR bacteria were stable (Table 1 (Tab. 1)). BSIs caused by CRKP was higher in the Oncology ICU – 6.38 (95% CI 3.78–10.15) per 10,000 patient days – when compared with wards with 0.39 (95% CI 0.21–0.69) or the stem-cell transplantation unit with 0.51 (95% CI 0.08–1.71) per 10,000 patient days. This can be a result of violating contact precautions with only two isolation rooms in a 8-bed ICU, since the consumption rate of alcohol-based hand antiseptics increased significantly in the entire Oncology Hospital from 55.41 (95% CI 53.17–57.72) liters per 1,000 patient days in 2019 to 94.23 (95% CI 91–97.54) liters per 1,000 patient days in 2020. 

The use of broad-spectrum antibiotics, such as carbapenems, piperacillin-tazobactam, polymyxins, amikacin, and glycopeptide antibiotics (vancomycin and teicoplanin), requires the approval of infectious-diseases (ID) specialists at Hacettepe University Hospitals; therefore, the patients with suspicion of MDR bacterial infection receive bedside consultation by ID specialists and are followed daily until the cessation of therapy, resolution of the symptoms, or discharge. Local diagnostic and management guidelines for empirical antibacterial treatment of common infections such as sepsis, pneumonia, intra-abdominal infections, urinary tract infections, and febrile neutropenia were prepared by a multidisciplinary team led by ID specialists, and were included in the hospital information management system operated by an internal server. Despite all these regulations, we experienced a significant increase in the consumption rates of antibiotics. The most significant increase was in meropenem consumption, which was the highest in COVID-19 ICUs. This could be related to prior use of fluroquinolones or ceftriaxone as initial treatment for suspected co-existing pulmonary infections in hospitalized COVID-19 patients (Table 4 (Tab. 4)) both in our hospital and in other treatment centers, whence critically ill patients were transferred to our COVID-19 ICUs, but we do not have data to support this hypothesis. On the other hand, pre-authorization of broad-spectrum antibiotics was not combined with audit and feedback in our hospital during the study period. Erturk et al. [18] reported that pre-authorization with feedback resulted in a decrease in the antibiotic consumption in a tertiary-care hospital with similar formal regulations. Increased rates of antibacterial consumption were reported from ICUs designed for COVID-19 patients as well as surgical patients during the COVID-19 pandemic [19], [20]. The consumption rates should be followed dynamically, as the pandemic is not stable, particularly during the emergence of new variants. Although a multicenter study from Brazil found an association between COVID-19 density and IDR of CRAB, they were not able to show any correlation between consumption rate of polymyxins and COVID-19 [14].

Our study is limited by its retrospective methodology, lack of molecular epidemiology for MDR bacteria, and being a single-center study. Moreover, we did not have patient-based information such as the source of BSI and impact of MDR on the patients’ outcome. Compliance with local antimicrobial treatment guidelines and rate of request for automatic stop orders for antibiotics were not monitored during the first year of the COVID-19 pandemic. 

In conclusion, BSIs caused by CRAB and ARE increased in the pandemic hospital, whereas IDRs of BSIs caused by other MDR bacteria were similar when compared with the pre-COVID era. The work overload and understaffing during the pandemic might have resulted in decreased compliance with infection prevention precautions, because there were no significant changes in the IDR of BSIs caused by MDR bacteria at the Oncology Hospital, which was designed to be COVID-19-free during the same period. The increased rates of antibiotic consumption despite ID authorization and local treatment guidelines is of concern. More attention should be paid to auditing and feedback components of the antimicrobial stewardship to avoid unnecessary use of broad-spectrum antibiotics instead of just relying on ID authorization.

## Notes

### Competing interests

Gökhan Metan received honoraria for speaking at symposia and lectures organized by Gilead Merck, Sharp, and Dohme (MSD). and Pfizer, as well as a consultation fee from the United Nations Turkey Office. He has also received travel grants from MSD, Pfizer, and Gilead to participate in conferences. Ömrüm Uzun received honoraria from Gilead for consulting. Serhat Ünal received honoraria for speaking at symposia from Pfizer, Gilead, and MSD. All other authors report no conflicts of interest.

### Acknowledgement

We thank the Infection Control Nurses of Hacettepe University Adult and Oncology Nurses for providing committee records for this study. We are grateful to all hospital staff for their valuable work in patient care during the COVID-19 pandemic. 

### Financial support

No financial support was provided for this study or article.

### Ethics approval

The study was approved by the Hacettepe University Non-Interventional Clinical Researches Ethics Board.

### ORCID ID

The ORCID ID of author Metan G is: https://orcid.org/0000-0002-2676-4557

## Figures and Tables

**Table 1:**
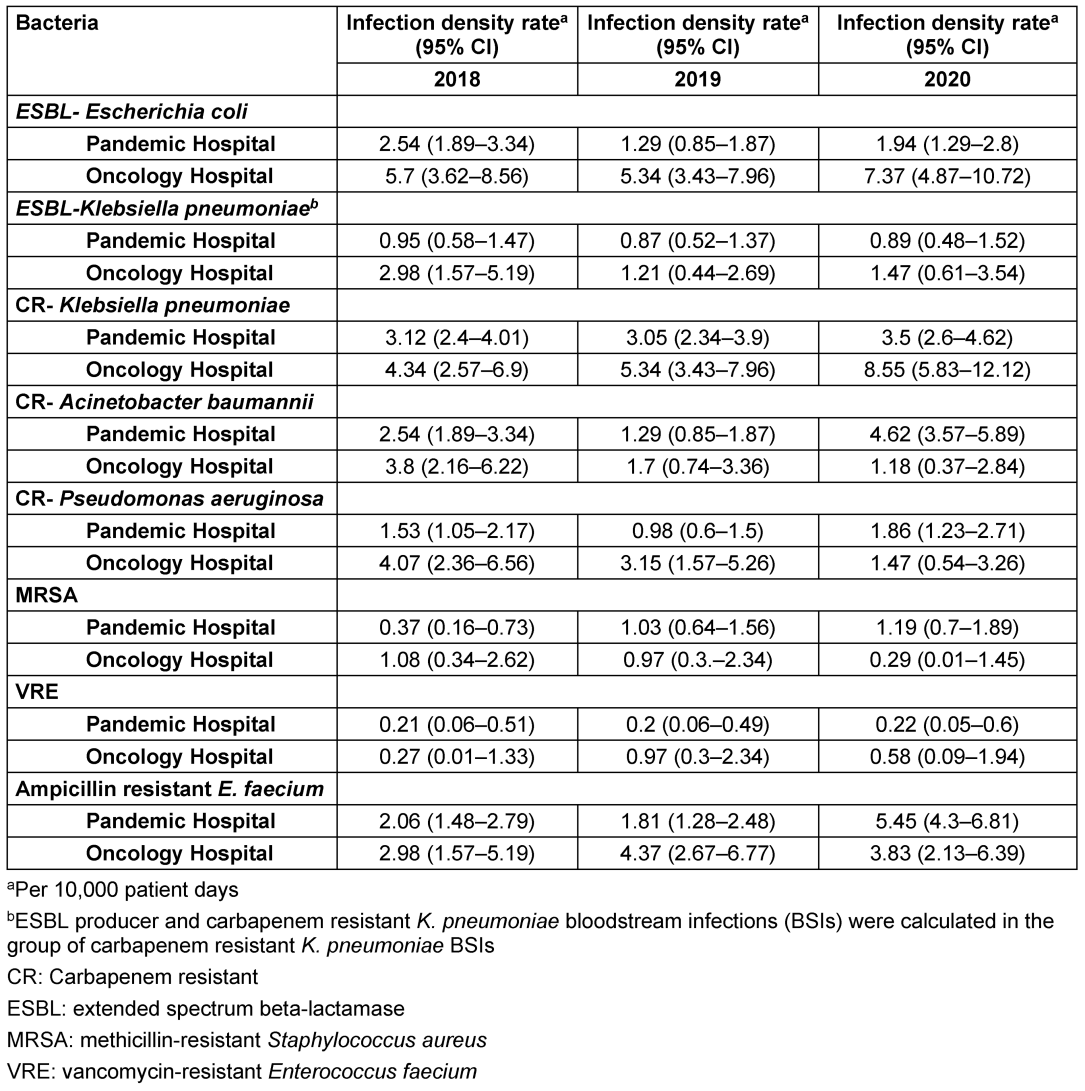
The infection density rate of nosocomial bloodstream infections caused by multidrug resistant bacteria at the Pandemic Hospital and Oncology Hospital

**Table 2:**
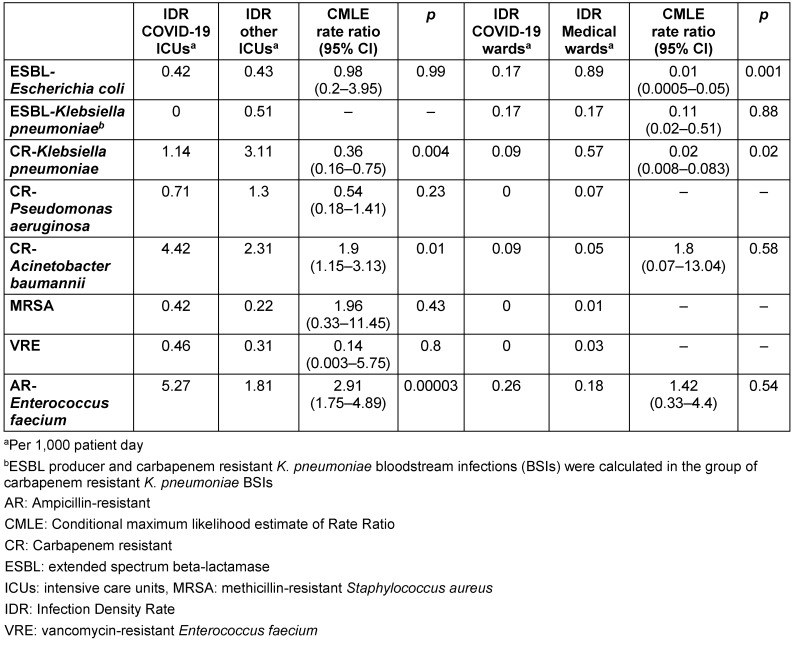
Comparison of the infection density rate of bloodstream infections caused by multidrug-resistant bacteria in COVID-19 intensive care units (ICUs) with other ICUs in the pandemic hospital and comparison of COVID-19 wards with other medical wards

**Table 3:**
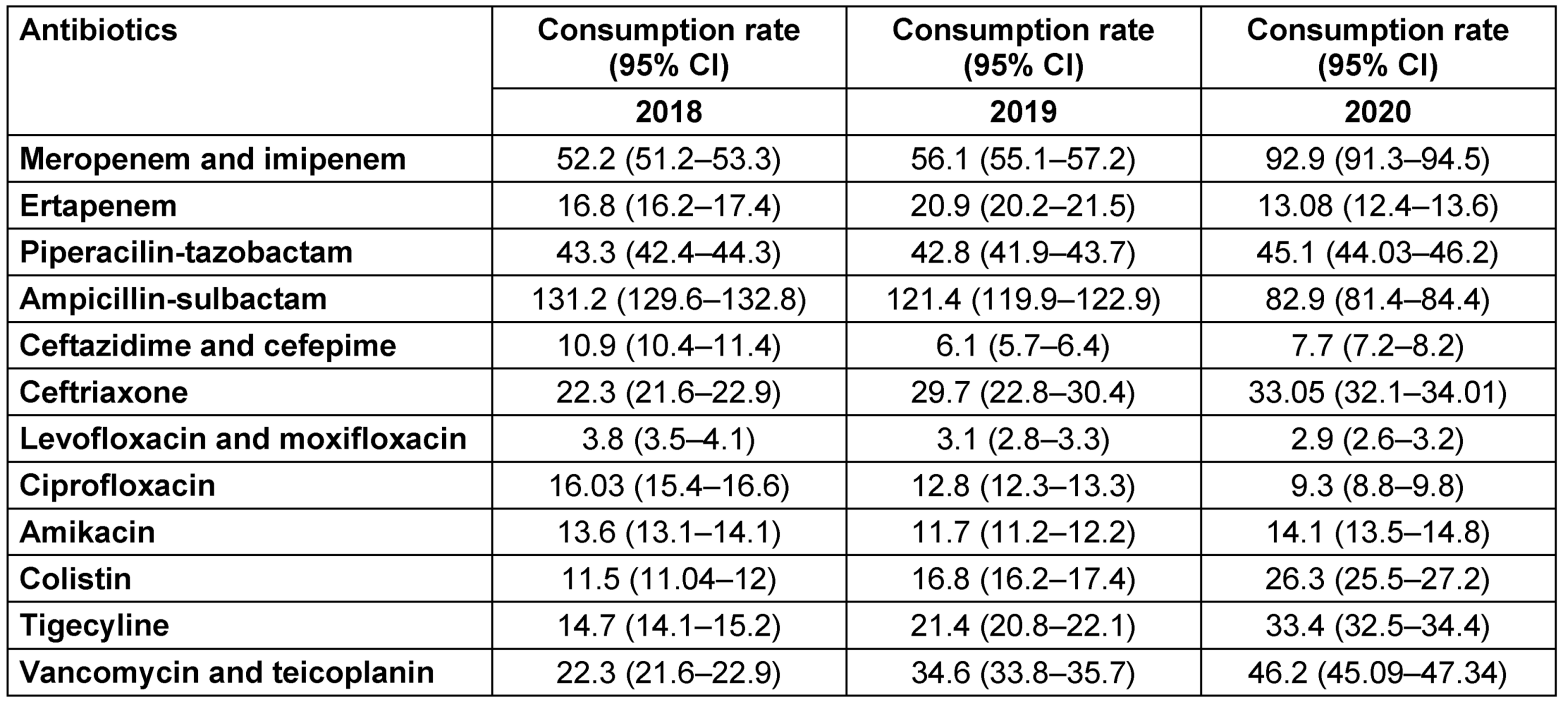
Consumption rates of antibiotics per 1,000 patient days in the pandemic hospital

**Table 4:**
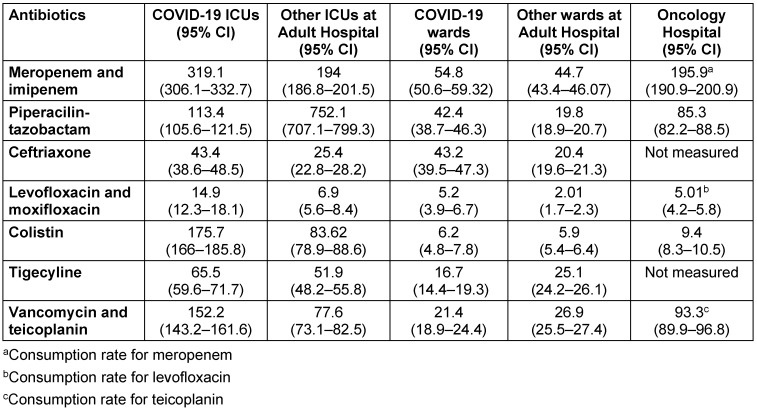
Comparison of the consumption rates per 1,000 patient days (95% Confidence interval, CI) of some broad-spectrum antibiotics at different clinics and hospitals, 2020
